# Effect of Beta-Cell Function on Glucose Variability When Switching From Insulin Degludec Plus a Dipeptidyl Peptidase-4 Inhibitor to Insulin Degludec/Liraglutide: Preliminary Results From a Pilot Study

**DOI:** 10.1155/ije/3911323

**Published:** 2025-12-02

**Authors:** Yuki Oe, Hiroshi Nomoto, Akinobu Nakamura, Saki Kuwabara, Yuka Takahashi, Ayano Yasui, Rimi Izumihara, Aika Miya, Hiraku Kameda, Kyu Yong Cho, Tatsuya Atsumi

**Affiliations:** ^1^Department of Rheumatology, Endocrinology and Nephrology, Faculty of Medicine and Graduate School of Medicine, Hokkaido University, Sapporo, Hokkaido, Japan; ^2^Division of Endocrinology, Metabolism, and Rheumatology, Department of Internal Medicine, Asahikawa Medical University, Asahikawa, Hokkaido, Japan

## Abstract

For patients with diabetes, achieving stable glucose control with minimal variability is highly significant. Incretin drugs have been shown to improve glycemic variability (GV), and we recently showed that a switch from a combination of a dipeptidyl peptidase (DPP)-4 inhibitor plus basal insulin to insulin degludec/liraglutide (IDegLira) improves GV, including the mean amplitude of glycemic excursions (MAGE), using continuous glucose monitoring in hospitalized patients with Type 2 diabetes. Here, we performed a secondary analysis to identify the factors related to an improvement in MAGE when switching to IDegLira. In patients with high MAGE, the change in C-peptide immunoreactivity during a glucagon test (ΔCPR) showed the highest correlation with the change in MAGE among the baseline patients' characteristics (*ρ* = −0.70; *p* < 0.05). Regression analysis confirmed a significant association between preserved CPR and amelioration of MAGE (*R*^2^ = 0.45; *p* < 0.05). Thus, this preliminary result implies that a treatment strategy that includes a glucagon-like peptide-1 receptor agonist might be beneficial for patients being treated with a DPP-4 inhibitor plus basal insulin who show high GV but have preserved beta-cell function.

**Trial Registration:**

University Hospital Medical Information Network (UMIN) Center Clinical Trials Registry: UMIN 000039460

## 1. Introduction

In diabetes mellitus, glycemic variability (GV) is increasingly recognized as a key determinant of clinical outcomes [1] for managing micro- and macrovascular complications [2]. Oxidative stress from high GV contributes to vascular endothelial dysfunction, resulting in the risk of cardiovascular events, secondary to atherosclerosis [1, 3, 4]. Minimizing GV could provide potential benefits in reducing oxidative stress, diabetic complications, and ultimately mortality [5–7]. Intermittently scanned continuous glucose monitoring (isCGM) is a useful means of determining the effects of antihyperglycemic agents on glucose fluctuations, and the factors affecting GV have been investigated in previous studies [8]. Recently, the usefulness of fixed-ratio combinations (FRCs) of medications has become apparent, and glucagon-like peptide-1 receptor agonists (GLP-1RAs) are a component of these combinations [9, 10]. Basal insulin primarily targets reductions in fasting plasma glucose (FPG), whereas long-acting GLP-1 receptor agonists modulate both fasting and postprandial glucose levels, thereby enhancing glycemic stability [11]. Therefore, an injectable FRC formulation comprising insulin degludec (IDeg) and the GLP-1RA liraglutide (IDegLira) would be expected to be effective, owing to the complementary actions of the two components.

Recently, we have shown that a switch from a combination therapy of dipeptidyl peptidase-4 (DPP-4) inhibitor plus a relatively low dose of basal insulin to IDegLira improves various indices of diurnal and daily GV, including the mean amplitude of glycemic excursions (MAGE) [12]. In Type 2 diabetes (T2D), individuals with very high MAGE are particularly vulnerable to the development of macrovascular and microvascular complications. [7] Therefore, identifying clinical factors related to better MAGE outcomes is important in order to determine which patients may achieve improved GV with an adjustment in treatment. In the present study, we aimed to identify the factors that contribute to an improvement in MAGE after switching to IDegLira in T2D patients with high MAGE.

## 2. Materials and Methods

### 2.1. Study Design and Participants

The present study comprised secondary analyses of data collected from our exploratory, prospective, single-arm, observational study, in which isCGM was used for hospitalized patients. The detailed rationale and protocol of the original study have been described previously [12]. Briefly, eligible participants were Japanese patients aged 20–80 years with T2D and glycated hemoglobin (HbA1c) ≥ 6.5%, who had been hospitalized at Hokkaido University Hospital and were recruited between February 2020 and April 2021. All the participants provided their written informed consent. Participants were instructed to maintain their prescribed dietary regimen and exhibited stable glucose levels for at least 3 consecutive days while administering their regular doses of DPP-4 inhibitor and IDeg (≤ 16 units/day). All the patients who met the criteria for enrollment underwent isCGM (FreeStyle Libre Pro sensor; Abbott Diabetes Care, Alameda, CA, USA) for up to 14 consecutive days. They remained on their original combination therapy for at least 2 days under monitoring and were subsequently switched to IDegLira at a dosage equivalent to their preceding IDeg treatment. After completing a 48-h transition, the subjects were observed for a further minimum of 2 days. Subsequently, measures of GV, including MAGE, were evaluated and compared between the periods of combination therapy and IDegLira. The protocol was approved by the Institutional Review Board of Hokkaido University Hospital Clinical Research and Medical Innovation Center (019-0293) and was performed in accordance with the ethical guidelines of the Declaration of Helsinki and all later modifications.

MAGE, which reflects GV within 1 day, has been used as an index for predicting patient outcomes [13–15]. Considering that patients with substantial GV are at high risk of complications [16] and MAGE > 3.3 mmol/L has been proposed as a suitable cutoff value for the risk of complications [4, 17], we performed a subanalysis of patients with high MAGEs (> 3.3 mmol/L) at baseline. The method used for the calculation of MAGE using isCGM data has been previously described [12]. The calculation of MAGE involved taking the arithmetic mean of differences between sequential peak and nadir glucose values, with inclusion restricted to excursions larger than 1 SD of the mean glucose concentration. For the evaluation of endogenous insulin secretion, fasting C-peptide immunoreactivity (CPR), C-peptide-to-glucose ratio (CPR index: CPI), the change in CPR during a glucagon test (ΔCPR), and the urinary C-peptide-to-creatinine ratio (UCPCR) were assessed at baseline. CPI reflects endogenous insulin secretion capacity and was calculated by dividing fasting CPR by FPG as follows: CPI = 100 × fasting CPR (ng/mL)/FPG (mg/dL) [18]. ΔCPR was calculated as the change in serum CPR between baseline and 6 min after the injection of 1 mg glucagon, conducted after stabilizing glucose concentration [19, 20]. UCPCR was calculated using the following formula: urinary C-peptide in fasting spot urine/spot urine creatinine.

### 2.2. Data Analysis

Normally distributed data were expressed as mean ± SD, and the rest were expressed as median (interquartile range). We used the paired *t*-test or the Wilcoxon signed-rank test for comparisons of pre- and postswitch data. Correlations were evaluated using Spearman's rank correlation analysis. Regression analysis was used to determine the effect of an explanatory variable on the change in MAGE associated with the switch to IDegLira. The model was confirmed using leave-one-out cross-validation (i.e., all participants except one were fit and predicted the out-of-sample participants' value). All the tests performed were two-tailed, and *p* < 0.05 was considered to represent statistical significance. Data were analyzed using GraphPad Prism 8.4.2 (GraphPad Software, Inc., San Diego, CA, USA) or JMP Pro 14.0.0 (SAS Inc., Cary, NC, USA).

## 3. Results

Among 75 patients with T2D who were admitted to our hospital during the study period, we obtained informed consent from 15 who met the inclusion criteria. Of these, the full set of isCGM data was available for 12 patients, and 10 had a high baseline MAGE (> 3.3 mmol/L) (Table 1). Therefore, data for these 10 patients were included in the present preliminary secondary analysis. Fifty percent of the participants were female, and their median age was 68.5 (57.3, 70.8) years. Their mean body mass index (BMI) and median duration of diabetes were 25.3 ± 3.2 kg/m^2^ and 5.0 (2.4, 17.3) years, respectively. Their FPG was well controlled, at 6.3 (5.9, 10.4) mmol/L, as part of our effort to improve their glycemic control while hospitalized. Of note, the glycemic control and body mass of the participants were stable at the time of the study, as shown previously [12]. All the participants had been undergoing treatment with a DPP-4 inhibitor plus IDeg before switching and were taking 8.0 ± 3.9 units IDeg/day (Tables 1 and 2).

**Figure 1:**
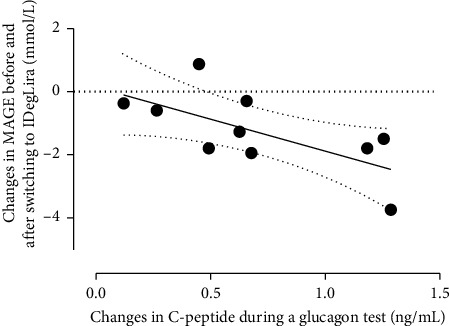
Regression analysis of the relationship between the change in C-peptide immunoreactivity during a glucagon test and the change in MAGE after switching to IDegLira. Each black circle represents a participant with a high baseline MAGE. The solid and dotted lines represent the regression line and the 95% confidence interval. MAGE, mean amplitude of glycemic excursions; IDegLira, insulin degludec/liraglutide.

The switch from a DPP-4 inhibitor plus IDeg to IDegLira significantly improved the MAGE of the participants (from 4.4 (4.0, 6.5) mmol/L to 3.8 (2.9, 4.6) mmol/L; *p* < 0.05), as well as their mean blood glucose concentration (from 6.4 (6.3, 8.1) mmol/L to 5.7 (5.5, 7.7) mmol/L; *p* < 0.01) (Supporting Table (available here)), and these findings were confirmed by the self-measurement of blood glucose concentration. We then performed correlation analysis to identify factors that were associated with the improvement in MAGE with the switch to IDegLira. As shown in Table 3, most of the baseline parameters did not show a clear correlation with the change in MAGE. Of the indices of beta-cell function assessed in this study, ΔCPR showed the highest correlation with the change in MAGE (*ρ* = −0.70; *p* < 0.05). Regression analysis showed that the insulin productivity was associated with an improvement in MAGE (change in MAGE = −0.0125 × ΔCPR + 0.4160; *R*^2^ = 0.45; *p* < 0.05) (Figure 1). The generalizability was confirmed by the leave-one-out cross-validation method. Importantly, ΔCPR did not correlate with other indices of endogenous insulin secretion, including fasting CPR, CPI, or UCPCR.

## 4. Discussion

This preliminary investigation demonstrated that T2D patients with high GV who switched from a DPP-4 inhibitor plus IDeg to IDegLira had an improvement in MAGE and that this improvement would depend on relatively intact beta-cell function, assessed using ΔCPR. We recruited patients whose baseline MAGE was > 3.3 mmol/L because this is the reported cutoff value for a risk of cardiovascular complications [16]. Patients with substantial GV despite adequate control of FPG would be suitable for such a treatment protocol, presumably leading to a better prognosis.

Both DPP-4 inhibitors and liraglutide have been shown to increase endogenous insulin secretion and improve glycemic control. However, to have this effect, many studies have shown that beta-cell function must be preserved [21–25]. In particular, a large-scale prospective study that investigated the effect of the initiation of a GLP-1RA (64% of the participants took liraglutide) on glycemic control showed a much lower glycemic response to the GLP-1RA in patients with severe insulin deficiency [21]. However, the doses of liraglutide used in these previous studies were relatively high (from 0.79 to 1.8 mg/day), and the participants had not previously been treated with DPP-4 inhibitors in most instances. In addition, the GVs of the participants were not compared in such low GLP-1RA component. The main finding of the present study is that beta-cell function might also be important for a beneficial effect of liraglutide treatment on GV, albeit when administered at a low dose (0.29 ± 0.14 mg/day) and even after treatment with a DPP-4 inhibitor. Considering that beta-cell function declines over time in patients with T2D [26], an appropriate early intervention aimed at preserving beta-cell function, such as the initiation of IDegLira, is important to minimize GV.

A strength of the present analysis is that we assessed several indices of beta-cell function, and as shown in Table 3, ΔCPR showed the highest correlation with the improvement in MAGE. ΔCPR is a clinically well-validated method of evaluating beta-cell function [19, 27]. Because glucagon directly stimulates insulin secretion from beta-cells [28], ΔCPR is a useful index of insulin secretory capacity. Of note, among several markers of beta-cell function that were assessed in a previous study, ΔCPR showed the closest correlation with human beta-cell area, implying that it would be useful for the assessment of responses to antidiabetic therapies [29]. In an earlier study, Takabe et al. reported that a higher ΔCPR was closely associated with enhanced early-phase insulin response during oral glucose tolerance tests in patients newly started on liraglutide [23]. Such an increase in the insulin response might contribute to a reduction in postprandial hyperglycemia, resulting in an improvement in MAGE. The reason why indices of beta-cell function other than ΔCPR did not show clear relationships with MAGE is presumably related to the study design. Most previous studies of the utility of indices of endogenous insulin secretion in a clinical setting were conducted in participants who were not administering insulin [30–33]. In contrast, in the present study, all the participants had been administered doses of IDeg that were sufficient to control FPG well, which might have partially inhibited endogenous insulin secretion. Limited to the race of Japanese, one previous cross-sectional study also showed that ΔCPR could be the clinical feature contributing to GV in patients treated by insulin [34]. Considering that switching from basal insulin therapy to IDegLira is a reasonable treatment option in a real-world clinical setting, ΔCPR would be an appropriate method of predicting the efficacy of such a switch.

The limitations of the original trial have been described previously [12]. The study was conducted over a relatively short period of time in a small number of hospitalized patients. In addition, the isCGM results were not blinded. Although eating habits and exercise intensity were stable owing to hospitalization, implying that participants could not change their lifestyle during the study period, our findings were limited owing to the preliminary study design. A longer-term randomized clinical trial with an adequate sample size should be conducted in the future to validate the present findings.

## 5. Conclusions

In patients with high MAGE, only ΔCPR, an index of endogenous insulin secretion, was associated with an improvement in MAGE. Treatment strategies involving FRC formulations that include a GLP-1RA might be beneficial for patients who have high GV but preserved insulin secretion and who are being treated with a DPP-4 inhibitor plus basal insulin. We expect that IDegLira will be effective in such patients, in whom it will help to reduce the risk of cardiovascular complications in the long term.

## Figures and Tables

**Table 1:** Participant characteristics.

Variables	High MAGE (MAGE > 3.3 mmol/L)	Low MAGE (MAGE ≤ 3.3 mmol/L)
	*n* = 10	*n* = 2
Age (years)	68.5 (57.3, 70.8)	49.0 ± 12.3
Female sex (*n*)	5	2
BMI (kg/m^2^)	25.3 ± 3.2	39.9 ± 18.1
Diabetes duration (years)	5.0 (2.4, 17.3)	0.5 ± 0.0
MAGE (mmol/L)	4.4 (4.0, 6.5)	55.3 ± 6.3
FPG (mmol/L)	6.3 (5.9, 10.4)	8.3 ± 2.4
HbA1c (%)	9.9 ± 2.7	9.7 ± 3.3
CPR (ng/mL)	1.93 ± 1.34	2.12 ± 0.49
CPI (ng/mL per mg/dL)	1.31 ± 0.82	1.53 ± 0.77
ΔCPR (ng/mL)	0.70 ± 0.42	1.97 ± 0.65
UCPCR (μg/g.Cre)	51.8 (28.0, 93.5)	36.6 ± 11.9
eGFR (mL/min/1.73 m^2^)	70.1 ± 16.2	79.6 ± 17.6
IDeg (unit/day)	8.0 ± 3.9	4.0 ± 0.0

*Note:* Values are expressed as mean ± SD or median (interquartile range). HbA1c, glycated hemoglobin; CPR, C-peptide immunoreactivity; ΔCPR, change in C-peptide immunoreactivity during the glucagon test; Cre, creatinine; IDeg, insulin degludec, before switching.

Abbreviations: BMI, body mass index; CPI, C-peptide index; eGFR, estimated glomerular filtration rate; FPG, fasting plasma glucose; MAGE, mean amplitude of glycemic excursions; UCPCR, urinary C-peptide/creatinine ratio.

**Table 2:** Concomitant drugs in the high MAGE population.

The proportion of oral hypoglycemic agent	*n* = 10
DPP-4 inhibitors (%)	10 (100.0)
Metformin (%)	7 (70.0)
SGLT2 inhibitors (%)	3 (30.0)
Alpha glucosidase inhibitors (%)	1 (10.0)
Glinides (%)	1 (10.0)
Insulin degludec (%)	10 (100.0)
Bolus insulin (%)	0 (0.0)
The proportion of antihypertensive drugs	
ARB/ACE inhibitors	3 (30.0)
CCB	2 (20.0)
Diuretic	2 (0.0)
The proportion of lipid-lowering agents	
Statins	6 (60.0)
Fibrates	1 (10.0)
The proportion of antiplatelet drug	3 (30.0)

*Note:* Values are expressed as number (%). DPP-4, dipeptidyl peptidase-4; SGLT2, sodium-glucose cotransporter 2; ARB, angiotensin II receptor blocker.

Abbreviations: ACE, angiotensin-converting enzyme; CCB, calcium channel blocker.

**Table 3:** Relationships between the change in MAGE associated with a switch to IDegLira and the baseline characteristics of the participants with high MAGE.

Variables	*ρ*	*p* value for Spearman's rank-correction
Age (years)	0.08	0.83
Gender (male = 1)	−0.31	0.42
BMI (kg/m^2^)	0.20	0.58
Diabetes duration (years)	−0.07	0.86
FPG (mmol/L)	−0.02	0.95
CPR (ng/mL)	−0.07	0.87
CPI (ng/mL per mg/dL)	0.08	0.84
ΔCPR (ng/mL)	−0.70	< 0.05
UCPCR (μg/g.Cre)	0.21	0.56
eGFR (mL/min/1.73 m^2^)	−0.60	0.07
IDeg (unit/day)	−0.21	0.57

*Note:* CPR, C-peptide immunoreactivity; ΔCPR, change in C-peptide immunoreactivity during a glucagon test; IDeg, insulin degludec before switching; IDegLira, insulin degludec/liraglutide.

Abbreviations: BMI, body mass index; CPI, C-peptide index; eGFR, estimated glomerular filtration rate; FPG, fasting plasma glucose; MAGE, mean amplitude of glycemic excursions; UCPCR, urinary C-peptide/creatinine ratio.

## Data Availability

The datasets used and/or analyzed during the current study are available from the corresponding author upon reasonable request.

## Supporting Information

Supporting Table. Parameters describing glucose variability in participants with a high MAGE treated with combination therapy or IDegLira.
