# Supplementary material for: Effect of Beta-Cell Function on Glucose Variability When Switching From Insulin Degludec Plus a Dipeptidyl Peptidase-4 Inhibitor to Insulin Degludec/Liraglutide: Preliminary Results From a Pilot Study
Source: Int J Endocrinol. 2025 Dec 2;2025:3911323. doi: 10.1155/ije/3911323 (PMC12688642; doi:10.1155/ije/3911323)
Supplement: Supporting Information — Additional supporting information can be found online in the Supporting Information section. [file 3911323.f1.docx]

Supplementary Table. Parameters describing glucose variability in participants with high MAGE treated with combination therapy or IDegLira

|  | IDeg + DPP4i | IDegLira | *P* value |
| --- | --- | --- | --- |
| MAGE (mmol/L) | 4.4 (4.0, 6.5) | 3.8 (2.9, 4.6) | < 0.05† |
| M-value (mmol/L) | 104.7 (103.0, 131.8) | 92.9 (89.4, 124.6) | < 0.01† |
| MAG (mmol/L) | 1.6 ± .0.4 | 1.3 ± 0.4 | < 0.01 |
| J-index | 6975.5 (6189.2, 12132.4) | 5216.5 (4837.8, 9505.5) | < 0.01† |
| CONGA (mmol/L) | 5.7 (5.5, 7.2) | 5.1 (4.9, 6.7) | < 0.01† |
| HBGI | 320.7 ± 42.9 | 295.1 ± 37.6 | < 0.01 |
| 24-h mean Glucose (mmol/L) | 6.4 (6.3, 8.1) | 5.7 (5.5, 7.7) | < 0.01† |
| CV (%) | 28.2 ± 8.5 | 23.3 ± 6.1 | 0.051 |
| SD (mmol/L) | 2.0 ± 0.9 | 1.5 ± 0.6 | < 0.01 |

Values are expressed as mean ± SD or median (interquartile range). *P* values are for IDeg + DPP-4i vs IDegLira. †Wilcoxon’s signed-rank test was applied to the following factors: MAGE, J-index, SD, TBR, TIR, and TAR. IDeg, insulin degludec; IDegLira, insulin degludec/liraglutide combination; DPP4i, dipeptidyl peptidase-4 inhibitor; MAGE, mean amplitude of glycemic excursions; MAG, mean absolute glucose; CONGA, continuous overall net glycemic action; HGBI, high blood glucose index; CV, coefficient of variation; SD, standard deviation.
